# Correction: Interaction between Age and Obesity on Cardiomyocyte Contractile Function: Role of Leptin and Stress Signaling

**DOI:** 10.1371/journal.pone.0105650

**Published:** 2014-08-11

**Authors:** The PLOS ONE Staff

The middle two panels of Figure 2 are incorrect. The authors have provided a corrected version of Figure 2 below.

**Figure 2:**
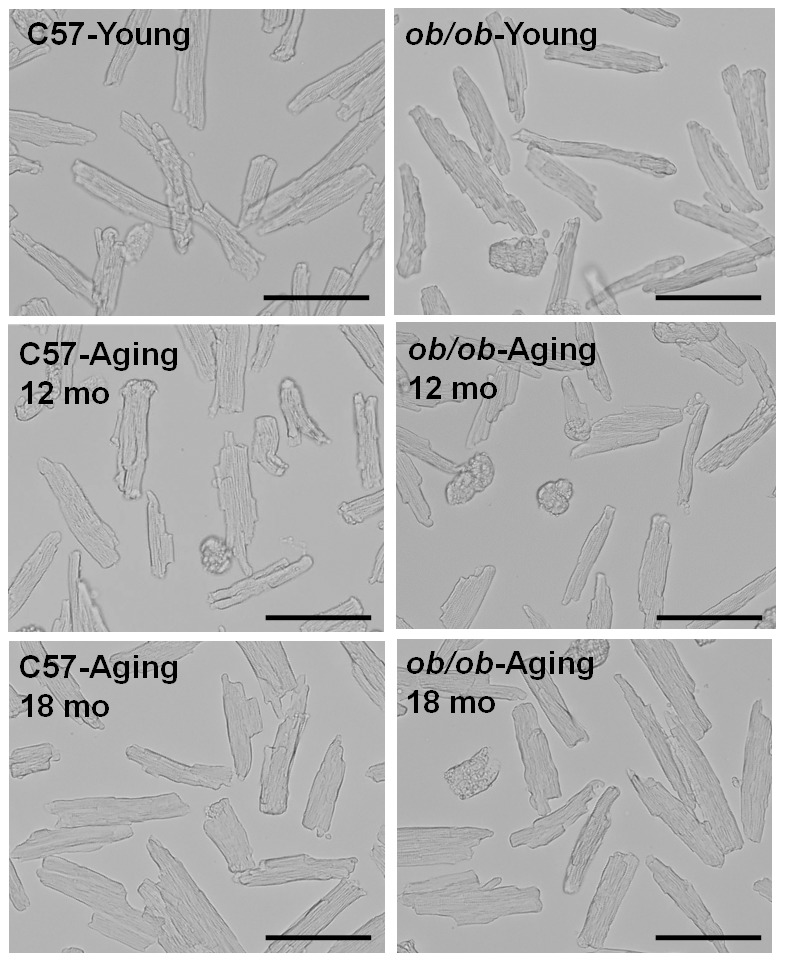
Light microscopic images of cardiomyocytes freshly isolated from young (4-month-old) and aging (12- or 18-month-old) lean (C57) and *ob/ob* mice. 200x, scale bar  =  100 µm.
